# Critical evaluation of (110) texture in lithium electrodeposits on isotropic Cu polycrystals

**DOI:** 10.1038/s41467-022-32949-y

**Published:** 2022-09-30

**Authors:** Chaojing Lu, Zongta Luo

**Affiliations:** 1 State Key Laboratory of Bio-Fibers and Eco-Textiles, Qingdao University, 266071 Qingdao, Shandong China; 2 College of Physics, Qingdao University, 266071 Qingdao, Shandong China

**Keywords:** Structure of solids and liquids, Batteries

**arising from** Q. Zhao et al. *Nature Communications* 10.1038/s41467-021-26143-9 (2021)

In their paper, Zhao et al.^1^ claimed enable to electrodeposit dominantly (110)-oriented lithium films on polycrystalline Cu foil of random orientation, and reported that the textured growth of metal Li electrode in battery enhances its cycling reversibility by regulating morphology. The crystallographic texture (preferred orientation) was quantified using the intensity fraction of 110 reflection in each *θ*–2*θ* scan of X-ray diffraction (XRD). There appear to be errors in methodology used for evaluating texture. Our interpretations on the XRD data don’t support the dominant (110) texture of lithium electrodeposits.

It is worth noting that the terminology of texture or preferred orientation is rarely seen in Zhao’s paper. The texture is instead described using various inappropriate phrases, including preferred crystal facet orientation, preferred crystal facets, crystal facet orientation, crystallographic facets, crystal facet, and crystallography.

The quantitation of (*hkl*) texture is measure of the volume fraction *f*_v_(*hkl*) of crystallites having the orientation (*hkl*) within the spread of ΔΩ. Zhao et al.^1^ claimed the Li electrodeposits of dominant (110) texture, meaning that the texture fraction *f*_v_(110) of each Li film exceeds at least 50%. Before the conclusion was drawn, they should quantify the *f*_v_(110) value of each Li film by calculating its crystallographic orientation distribution function (ODF). For this purpose, X-ray pole figure combined with electron diffraction (ED) and transmission electron microscopy (TEM) could be the preferred method^2–7^. In Zhao’s paper, unfortunately, neither X-ray pole figure nor TEM/ED evidence is available. Instead, Zhao et al.^1^ preferred *θ*–2*θ* scan XRD, which cannot give much information about ODF.

Based on the *θ*–2*θ* scans in Fig. S3 of their paper, Zhao et al.^1^ emphasized the high intensity-fractions of 110 peak ranging from 88.3% to 91.2% (see supplementary Table 1 in Zhao’s paper) and then draw the conclusion of dominant (110) texture in their Li electrodeposits. Actually, they quantified (110) texture using the intensity fraction ${p}_{110}=\frac{\Sigma {I}_{{hh}0}}{\Sigma {I}_{{hkl}}}$^8^$,$ here *I*_*hkl*_ denotes the measured integrated intensity of *hkl* peak. From PDF #15-0401 for lithium powder, we know ${I}_{110}^{*}$/${I}_{200}^{*}$/${I}_{211}^{*}$ ≈ 100/30/40. Then we obtain ${p}_{110}^{*}$ of ≈51.5−58.8% for Li powder depending on the number of *hkl* peaks used, much higher than the expected ${f}_{{{{{{\rm{v}}}}}}}^{*}(110)$ value of ≈2.3% for Li powder when the maximum tilt deviation angle *ψ*_max_ of (110) orientation is 5°. Here

1 $${f}_{{{{{{\rm{v}}}}}}}^{*}\left(110\right)={m}_{110}\frac{\Delta \Omega }{4{{\pi }}}=6{{\times }}(1-{{{{{\rm{cos }}}}}}{{{\psi }}}_{{{{{{\rm{max }}}}}}}),$$

where *m*_110_ = 12 is the multiplicity factor of {110} planes, ΔΩ is the solid angle of a spherical crown within the *ψ*_max_ and 4*π* the solid angle of the pole spherical surface. Therefore, it is totally wrong to quantify (110) texture with the intensity fraction ${p}_{110}$. As we know, it is unreliable and incorrect to make any quantitative assessment of (110) texture with the Lotgering degree of orientation ${f}_{110}=\frac{{p}_{110}-{p}_{110}^{*}}{1-{p}_{110}^{*}}$^9–11^$.$ Such indiscriminate assessments give usually an overestimated value of texture^4–6,9^. Quantifying (110) texture with the ${p}_{110}$ is even stray farther away, see Table 1.

**Table 1:** Comparison of the intensity fraction *p*_110_ and the Lotgering degree of orientation *f*_110_ as well as the possible texture fraction *f*_v_(110) of the Li electrodeposits estimated from the intensity ratio *I*_200_*/I*_211_*/I*_110_ in their *θ*−2*θ* scans

Sample	Data reported in Zhao’s paper				*f*_110_	Possible *f*_v_(110) values estimated within ΔΩ		
	*D* (μm)	*t* (μm)	*I*_200_*/I*_211_*/I*_110_	*p*_110_		Δ*ψ*Δ*ω* ≈ 6° × 1.5°	*ψ*_max_ = 5°	*ψ*_max_ = 10°
5 mAh Li deposited	2.7	35	61/71/1000	88.3%	71.6%	1.10%	11.1%	34.7%
10 mAh Li deposited	4.8	56	27/79/1000	90.4%	76.7%	1.37%	13.5%	39.9%
20 mAh Li deposited	6.4	108	25/72/1000	91.2%	78.6%	1.49%	14.5%	41.9%
Li powder	—	—	30/40/100	58.8%	—	0.21%	2.3%	9.12%

The corresponding data for Li powder are listed for comparison and its *I*_200_*/I*_211_*/I*_110_ values are known from PDF #15-0401. The average surface grain size *D* of each Li deposit and its thickness *t* are also listed. The possible *f*_v_(110) values within different orientation spreads ΔΩ are estimated by using Eq. (2). The angle window of diffractometer ΔΩ_w_ = *π*Δ*ψ*Δ*ω*/4, here Δ*ψ* and Δ*ω* in unit of radian are the diameters of angle window parallel and perpendicular to the diffraction plane, respectively^12,13^. The possible Δ*ψ*Δ*ω* value of ≈6° × 1.5° is a typical angle window of X-ray diffractometer with a conventional point detector. The spread range of (110) texture ΔΩ = 2*π*(1 − cos*ψ*_max_), where *ψ*_max_ is the maximum tilt deviation angle of (110) orientation. The *ψ*_max_ is usually given to be 5° and it can be customized based on the requirement. The possible *ψ*_max_ of 10° is large enough for quantifying the (110) texture of Li films because for Li powder the ${f}_{{{{{{\rm{v}}}}}}}^{*}\left(110\right)$ within the spread ΔΩ reaches a high value of 9.12%. Considering all the values of the interplane angles ∠{*hkl*}:(110) for cubic Li, we know that each Li crystallite with any (*hkl*) orientation has always a set of (110) lattice planes oriented with a tilt angle *ψ* of ≤30° or 45°. The orientation spread ΔΩ is an essential parameter in the quantitation of texture fraction *f*_v_(*hkl*) and thus the *ψ*_max_ value customized needs be indicated when each *f*_v_(*hkl*) value is reported.

In a symmetric *θ*–2*θ* scan the lattice planes contributing to reflection *I*_*hkl*_ are all oriented parallel or nearly parallel to the film surface. Only a subset of grains is monitored, for which the plane normal lies in an angle window ΔΩ_w_ around the substrate normal, when ΔΩ_w_ characterizes the divergence of the X-ray beam received by the point detector. Here, ΔΩ_w_ ≈ *π*Δ*ψ*Δ*ω*/4, where Δ*ψ* and Δ*ω* in unit of radian are the diameters of angle window parallel and perpendicular to the diffraction plane, respectively^12,13^. If the ΔΩ_w_ covered the tilt spread ΔΩ of (110) texture, the *f*_v_(110) values of the Li electrodeposits could be roughly estimated from the intensity ratio of the 110 peak to a nearby peak *hkl*^11^. In order to balance the intensity fluctuation of weak 200 and 211 peaks, their total intensity is chosen to represent the diffraction contributions from the randomly oriented Li component. From Equation (10) in ref. 11. one can know $\frac{{I}_{200}+{I}_{211}}{{I}_{110}}{{\propto }}\frac{1-{f}_{{{{{{\rm{v}}}}}}}(110)}{{f}_{{{{{{\rm{v}}}}}}}(110)}$. Similarly, we can write $\frac{{I}_{200}^{*}+{I}_{211}^{*}}{{I}_{110}^{*}}{{\propto }}\frac{1-{f}_{{{{{{\rm{v}}}}}}}^{*}(110)}{{f}_{{{{{{\rm{v}}}}}}}^{*}(110)}$ for powder. Division of the two formulas yields

2 $$\frac{{I}_{200}+{I}_{211}}{{I}_{110}}\frac{{I}_{110}^{*}}{{I}_{200}^{*}+{I}_{211}^{*}}\frac{1-{f}_{{{{{{\rm{v}}}}}}}^{*}(110)}{{f}_{{{{{{\rm{v}}}}}}}^{*}(110)}=\frac{1-{f}_{{{{{{\rm{v}}}}}}}(110)}{{f}_{{{{{{\rm{v}}}}}}}(110)}.$$

Supposing that Δ*ψ*Δ*ω* ≈ 6° × 1.5°, which is a typical possible angle window of diffractometer^12^, we can calculate ${f}_{{{{{{\rm{v}}}}}}}^{*}\left(110\right)={m}_{110}\frac{\Delta {\Omega }_{{{{{{\rm{w}}}}}}}}{4{{\pi }}}=\frac{3}{4}\Delta {\psi }\Delta {\omega }\,{{\approx }}\,0.21\%$. Then the possible *f*_v_(110) values are estimated to be from 1.10% to 1.49% based on Eq. (2), see Table 1. Obviously, the *θ*–2*θ* scans with high ${p}_{110}$ of 88.3%–91.2% could come from the Li deposits of slight (110) texture, further confirming that it is incorrect to quantify (110) texture with ${p}_{110}$. It is easily understandable that the intensity ratio $\frac{{I}_{110}}{{I}_{200}+{I}_{211}}$ increases from 100/70 to 1000/79 as long as the *f*_v_(110) value rises from 0.21% to 1.49% when the angle window ΔΩ_w_ covers the texture spread ΔΩ. For  evaluating the real (110) texture of each Li deposit, nevertheless, we have to know its ODF.

Generally, a two-dimensional (2D) XRD pattern provides much more information about ODF than a *θ*−2*θ* scan. The texture can be derived from analysis of the intensity variations along Debye-Scherrer rings^7^. Unfortunately, the 2D patterns shown in Zhao’s paper present a limited sector of the homogeneous diffraction rings without noticeable intensity change. This fact suggests intuitively that the Li deposits might be of nearly random orientation with slight (110) texture. This possibility would be more reasonable if some diffractometer-specific intensity corrections are considered when the *f*_v_(110) values are estimated using Eq. (2). For example, the relative intensity of 110 peak from film sample increases significantly at lower 2*θ* angles due to the increment of diffraction volume, whereas such effect is generally very weak in the case of powder sample. Please note that the penetration thickness *t*_0_ of Cu *Kα* radiation in metal Li is ≈2.63 cm, much thicker than the Li film thicknesses of 35−108 μm.

Meanwhile, we need consider another possibility that the (110) texture is so scattered that the intensity variations along the Debye rings occur beyond the sector available, see our analysis in Fig. 1, although the well-distributed intensity along the 110 ring in Fig. 1e disagrees with the gradual intensity change in usual textured cases. In this possible case, the absence of intensity variation along the weak 211 ring within |*χ*| ≤ 5° demonstrates that the tilt spread Δ*ψ* of (110) texture should be ≤15°. This means that the 2D detector employed is large enough for collecting the intensity variations along the Debye rings. We encourage Zhao et al.^1^ collect two 2D frames with either 110 or 211 ring in the middle of the 2D detector, and then merge them into a diffraction pattern^7^. The homogeneous 110 ring in Fig. 1e might indicate that the Li deposit has well-distributed pole density within the ${{\psi }}_{{{{{{\rm{max }}}}}}}$ of 10°. In this possible case, we could calculate ${f}_{{{{{{\rm{v}}}}}}}^{*}\left(110\right)$ using Formula (1) and then estimate the possible *f*_v_(110) values with Eq. (2), see Table 1. At this moment, the estimated *f*_v_(110) values of 34.7%–41.9% may not be true because no ODF of any Li deposit can be unambiguously derived from the 2D patterns in Zhao’s paper.

**Fig. 1:**
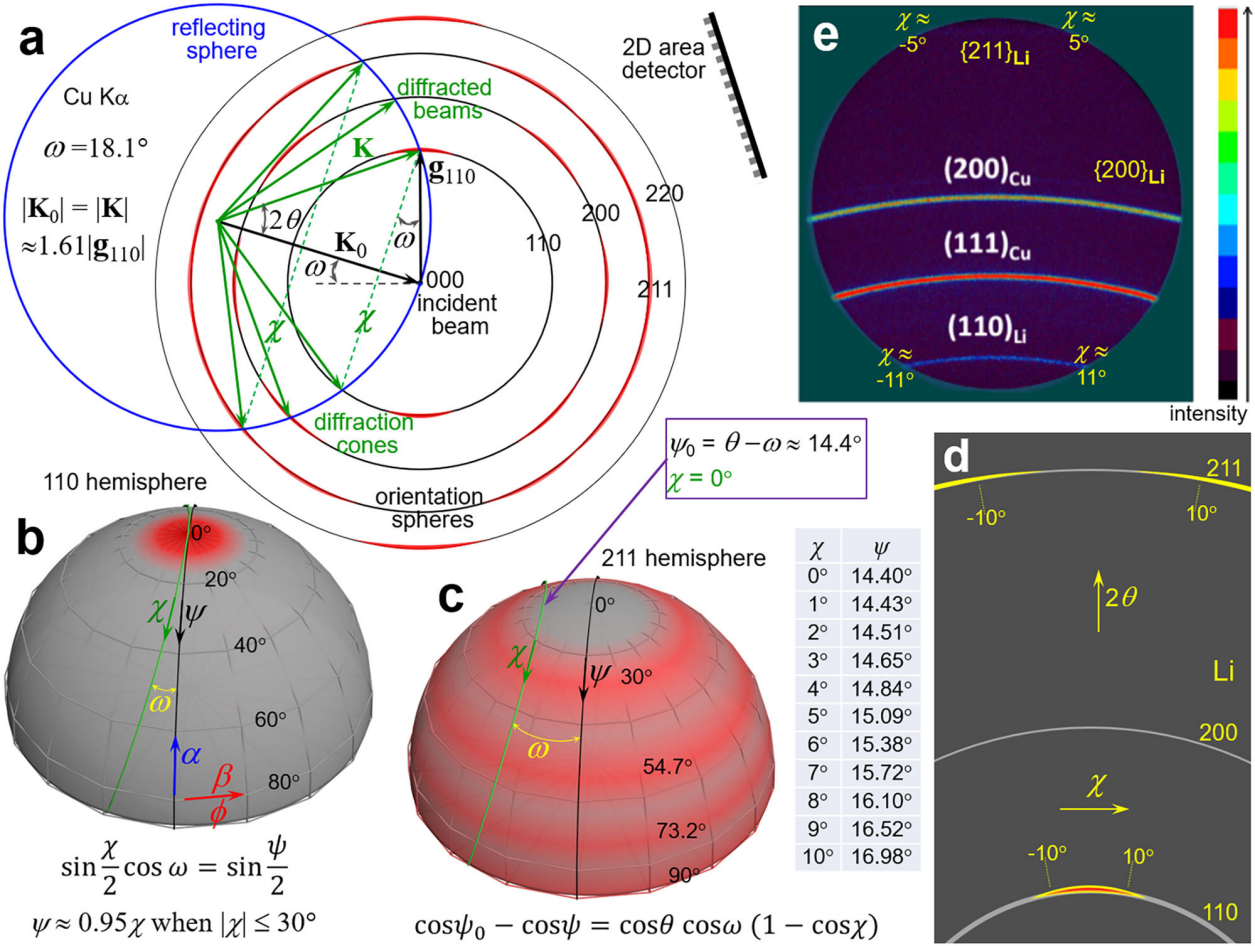
XRD analyses of crystallographic orientation distribution in deposited lithium on isotropic polycrystalline Cu. **a**–**d** Schematic drawings in reciprocal space to show the geometry requirement producing diffraction with intensity variation from polycrystal Li, which is a mixture of randomly oriented grains and (110)-textured grains with the tilt spread Δ*ψ* of ≤15°. **a** Superimposing the reciprocal lattice of a dominantly (110)-textured polycrystal Li on the reflecting sphere construction demonstrates the geometric conditions producing Debye diffraction cones of intensity variation. A set of homocentric spherical surfaces display raised red spherical crowns or broad latitude-zones denoting enhanced orientation density due to the scattered (110) texture. They intersect with the reflecting sphere at a set of coaxial circles *χ* representing the allowed Bragg reflections. The incident angle *ω* ≈ 18.1° was told by the first author Zhao. **b** {110} orientation hemisphere showing the tilt scatter of (110) fiber texture and the relation between the tilt angle *ψ* and the diffraction azimuth *χ*. The gray denotes well-distributed random orientation of low pole density while the red arctic area the scattered texture of enhanced pole density. **c** {211} orientation hemisphere showing the broadenings and overlapping of four latitude-zones centered at *ψ* = 30°, 54.74°, 73.22°, and 90°, corresponding to the interplane angles ∠{211}:(110). The *χ*–*ψ* relation indicates that *ψ* shifts from 14.84° to 15.09° with increasing *χ* from 4° to 5°. The latitude-zone centered at *ψ* = 30° with Δ*ψ* of ≥15° can broaden to the position of *ψ* = 15°. Thus, (211) diffraction intensity enhances when *χ*  ≥ 5° due to the contribution of near-(110)-oriented grains. **d** Schematic 2D XRD pattern from polycrystalline Li of dominant (110) texture. The weak rings come from Li grains of random orientation while the strong spots elongated along the 110 and 211 rings from near-(110)-oriented grains. The two yellow spots elongated along the 211 ring would contact and even overlap when the tilt spread Δ*ψ* ≥ 15.6°. **e** 2D XRD pattern of a Li film deposited on Cu foil with an areal capacity of 20 mAh cm^−2^, reproduced from Fig. 2d in Zhao’s paper. The Li(110) ring presents homogeneous intensity while both the Li{200} and Li{211} rings are weak.

In summary, it is incorrect to quantify (110) texture with the intensity fraction ${p}_{110}$. The XRD data in Zhao’s paper are insufficient to support the Li electrodeposition of dominant (110) texture. Zhao et al.^1^ need provide new convincing evidences to prove their claim of dominant (110) texture in the electrodeposited Li films on isotropic Cu polycrystals.

## Data Availability

The data that support the findings of this study are available from the corresponding author (Prof. Chaojing Lu) upon reasonable request.

## Code availability

No custom code or mathematical algorithm is used in the manuscript.
